# Modeling the effect of surgical sterilization on owned dog population size in Villa de Tezontepec, Hidalgo, Mexico, using an individual-based computer simulation model

**DOI:** 10.1371/journal.pone.0198209

**Published:** 2018-06-01

**Authors:** Luz Maria Kisiel, Andria Jones-Bitton, Jan M. Sargeant, Jason B. Coe, D. T. Tyler Flockhart, Erick J. Canales Vargas, Amy L. Greer

**Affiliations:** 1 Department of Population Medicine, Ontario Veterinary College, University of Guelph, Guelph, Ontario, Canada; 2 Centre for Public Health and Zoonoses, University of Guelph, Guelph, Ontario, Canada; 3 Department of Integrative Biology, University of Guelph, Guelph, Ontario, Canada; 4 Rabies and Zoonoses Prevention Program, Servicios de Salud de Hidalgo, Mineral de la Reforma, Hidalgo, Mexico; Faculty of Animal Sciences and Food Engineering, University of São Paulo, BRAZIL

## Abstract

Surgical sterilization programs for dogs have been proposed as interventions to control dog population size. Models can be used to help identify the long-term impact of reproduction control interventions for dogs. The objective of this study was to determine the projected impact of surgical sterilization interventions on the owned dog population size in Villa de Tezontepec, Hidalgo, Mexico. A stochastic, individual-based simulation model was constructed and parameterized using a combination of empirical data collected on the demographics of owned dogs in Villa de Tezontepec and data available from the peer-reviewed literature. Model outcomes were assessed using a 20-year time horizon. The model was used to examine: the effect of surgical sterilization strategies focused on: 1) dogs of any age and sex, 2) female dogs of any age, 3) young dogs (i.e., not yet reached sexual maturity) of any sex, and 4) young, female dogs. Model outcomes suggested that as surgical capacity increases from 21 to 84 surgeries/month, (8.6% to 34.5% annual sterilization) for dogs of any age, the mean dog population size after 20 years was reduced between 14% and 79% compared to the base case scenario (i.e. in the absence of intervention). Surgical sterilization interventions focused only on young dogs of any sex yielded greater reductions (81% - 90%) in the mean population size, depending on the level of surgical capacity. More focused sterilization targeted at female dogs of any age, resulted in reductions that were similar to focusing on mixed sex sterilization of only young dogs (82% - 92%). The greatest mean reduction in population size (90% - 91%) was associated with sterilization of only young, female dogs. Our model suggests that targeting sterilization to young females could enhance the efficacy of existing surgical dog population control interventions in this location, without investing extra resources.

## 1. Introduction

The overabundance of dogs in developing countries poses significant public health, animal health, and animal welfare concerns [1–3]. Reproduction control is one of several methods than can be used for controlling the growth of dog populations [3]. Surgical sterilization is the most common type of dog reproduction control and remains the most frequently performed pet contraception procedure in veterinary practice [4]. Surgical sterilization can not only help to limit the increase of the dog population by preventing the birth of unwanted puppies [3], but offers other benefits including the prevention of some canine diseases such as mammary neoplasia or benign prostatic hyperplasia [4–8]. Furthermore, surgical sterilization can have an impact in dog’s unwanted sexual behaviors such as a reduction in roaming, mounting, urine marking, and sexual aggression, when performed in young animals (6–16 weeks) [7]. While surgical sterilization is commonly used in developed countries, the high cost and limited resources (including veterinary surgeons) in many developing countries makes surgical sterilization an infeasible method of population control for large-scale application [9].

In Mexico, the Ministry of Health started a subsidized pet (owned dogs and cats), high volume, surgical sterilization pilot program in 1994. This program was implemented as a result of the growing number of pets participating in the yearly national rabies vaccination program in the country [10]. In an effort to control the rising costs of the subsidized rabies vaccination program [10], a focus was placed on surgical sterilization of owned dogs in order to reduce the number of annual vaccine doses that would need to be administered over time. While this was the primary reason for the implementation of the sterilization program, reducing the number of owned dogs via sterilization could also create opportunities for unowned dogs to be re-homed when homes become available, hence balancing the supply and demand of dogs. Currently in Mexico, the pet sterilization program is offered in all of the 32 states and has become the public policy for controlling the owned pet population in this country [10]. Nevertheless, local health authorities in Mexico have limited funds and resources to address and control the pet population, especially the large population of owned dogs that remain the most popular pet in several parts of Mexico [11–13]. For this reason, dog population control programs in Mexico need to be carefully designed to not only have high intervention effectiveness but also be good value for money.

Mathematical models and computer simulation can evaluate various intervention strategies, with varying degrees of effectiveness, without incurring the actual cost of implementation. The use of simulation models can therefore help identify how to best target and optimize the limited resources that are available for dog population control measures. Several mathematical models have been developed to evaluate the effect of population control interventions such as sterilization and euthanasia in dog populations (both stray and owned) [14–19]. Different mathematical approaches have been used for the development of such models including, basic demographic equations [14], differential equations [15–18], and agent base simulation [19].

Villa de Tezontepec is one of the 84 municipalities in the state of Hidalgo, in central-eastern Mexico. The availability of detailed empirical data on the owned-dog population in this community and data from the government subsidized sterilization program made it an ideal case study for an evaluation of the owned dog population demographics and the projected long-term effect of the subsidized sterilization program using a simulation model. The municipality has an estimated human population of 11,746, [20]. Kisiel et al. [13] reported that Villa de Tezontepec had an estimated human: owned dog ratio of 3.4:1 and that approximately two-thirds (65%) of the households in the municipality owned one or more dogs. Approximately 74% of the owned dogs were older than one year, and only 45% of the owned dogs were kept confined when unsupervised. Fifty-five percent of the dogs were unconfined totally or partially during a 24-hour period. Surgical sterilization was more common in female dogs (37%) than male dogs (14%). Out of all reported surgically altered dogs, 80% were sterilized during subsidized government surgical sterilization clinics. At the time of writing, the authors of the study identified the government surgical sterilization program within this region used a “mixed” approach where both young dogs (sexually immature) and adult dogs (sexually mature) were sterilized at an average frequency of 21 surgeries per month (8.6% annually), with no specific preference for younger or adult dogs or sex. For our purposes, surgical sterilization refers to both the sterilization of female and male dogs.

Using a stochastic, individual-based model, informed by empirical data collected in Villa de Tezontepec, Hidalgo Mexico [13], the objective of this study was to compare the projected impact of surgical sterilization interventions that focused differentially on dogs of different ages and sexes, and also considered different levels of surgical capacity, on the number of owned dogs. The model outcome of interest was the total owned dog population size over a 20-year time period. Only owned dogs were modeled and non-owned dogs were not included.

## 2. Materials and methods

### 2.1 Model description

A stochastic, individual-based model was constructed using Anylogic, University, 7.2.0 (XJ Technologies). The model framework was based on the development of a hypothetical population (*in silico*) of owned male and female dogs in Villa de Tezontepec, Hidalgo. “Owned” dogs were defined as those dogs for which someone claims some right over or states that they are their property [21]. The hypothetical population of dogs included dogs that were confined (not permitted to roam-freely during a 24-hour period when unsupervised) and dogs that were unconfined (allowed by their owners to roam-free and unsupervised, totally or partially during a 24-hour period) as per Kisiel et al., 2016 [13]. The model was not spatially specific. The individual components of the model (females and male dogs) did not interact spatially in the model.

### 2.2. Hypothetical model population structure

**Fig 1:**
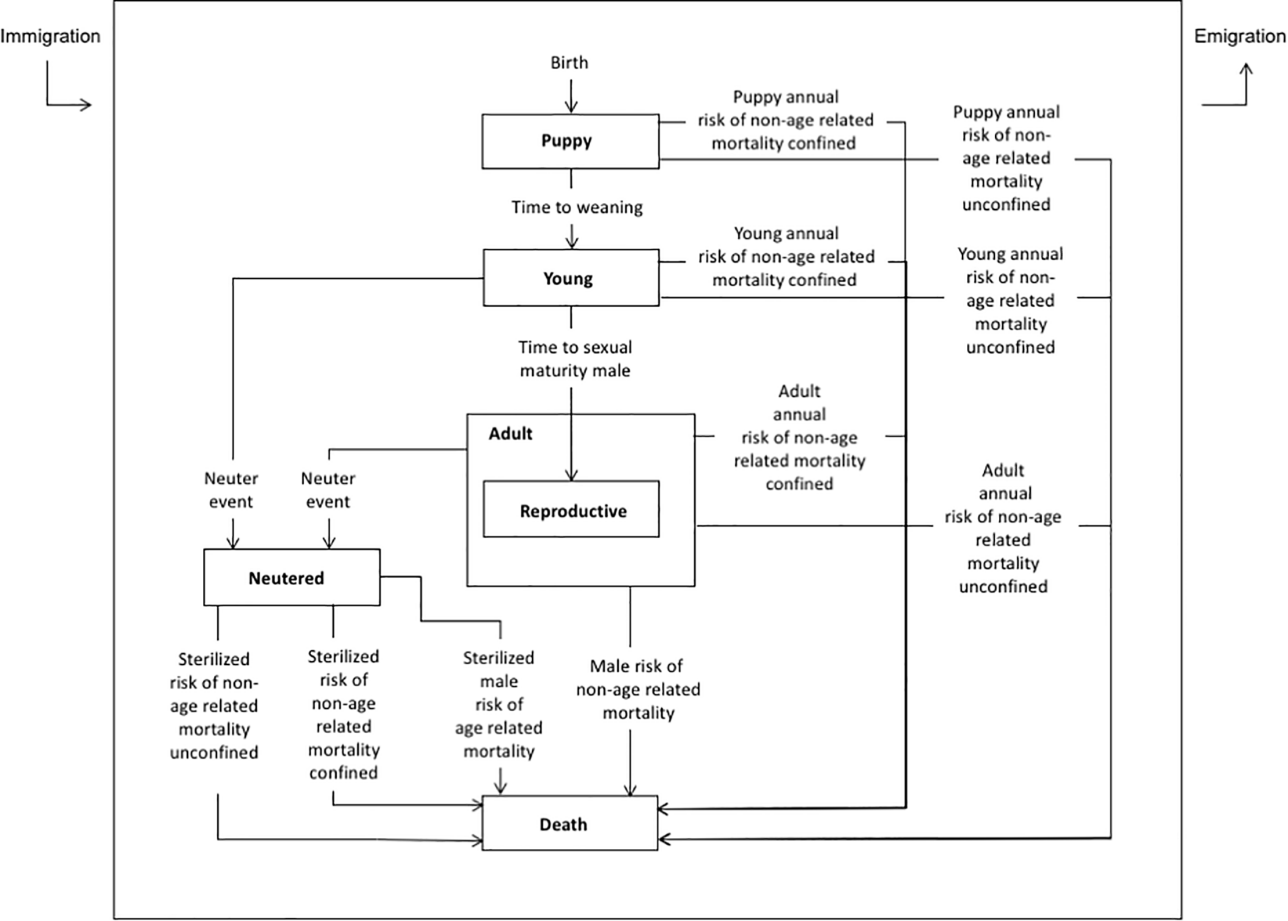
State chart for male dogs describing the individual life states and transitions, as well as immigration and emigration, in the individual-based model evaluating owned dog population control interventions in Villa de Tezontepec, Hidalgo Mexico.

**Fig 2:**
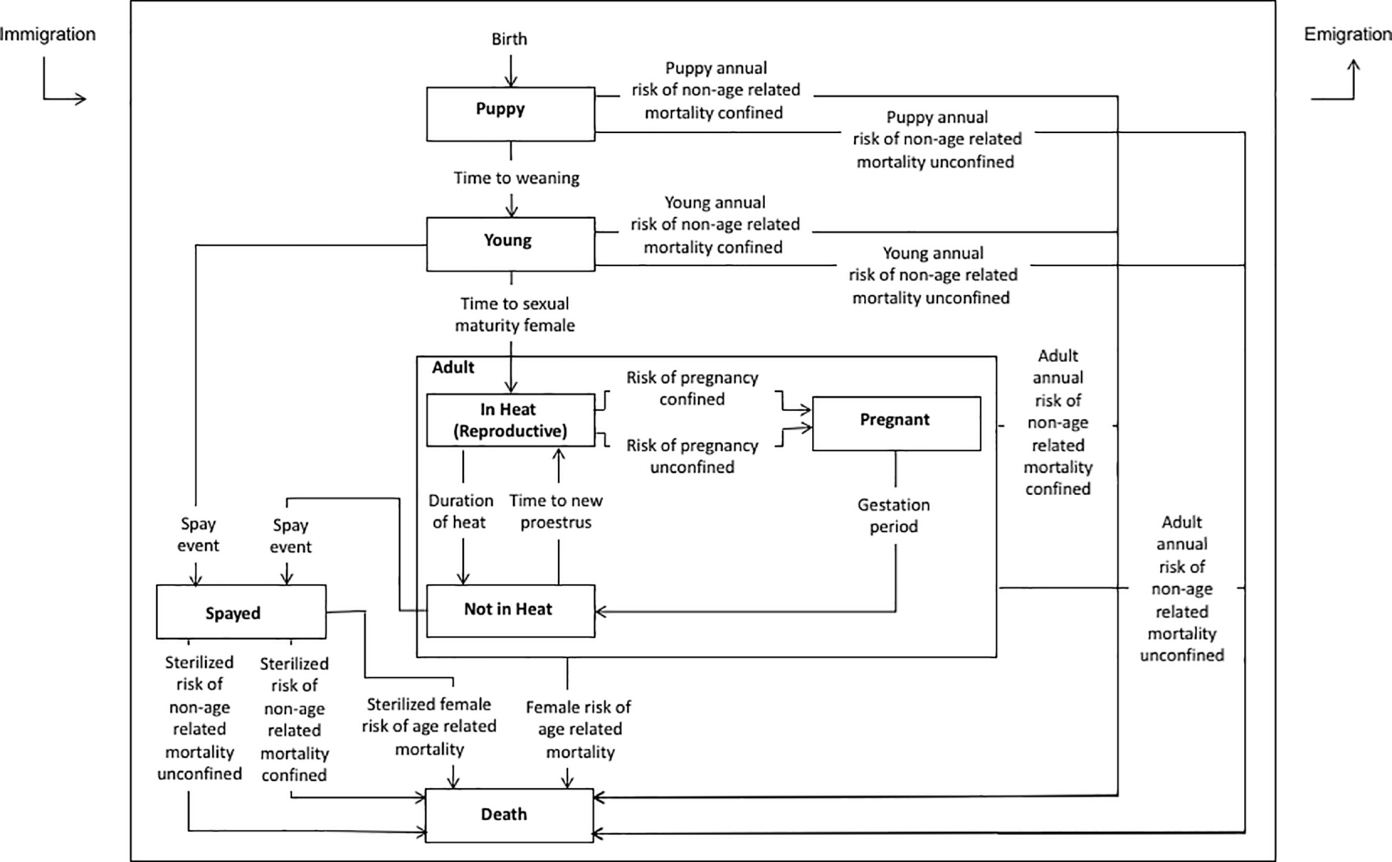
State chart for female dogs describing the individual life states and transitions, as well as immigration and emigration, in the individual-based model evaluating owned dog population control interventions in Villa de Tezontepec, Hidalgo Mexico.

The individual entities in the model represented female and male dogs. The initial hypothetical population of dogs consisted of 2924 dogs (1222 female dogs and 1702 male dogs), based on observed dog ownership data from the region [13], specifically related to the number of households owning dogs, and the mean number of dogs owned per household. This dog population was used to establish an upper limit of community capacity for owned dogs. This community capacity represented the maximum number of dogs that were owned by the current dog-owning households in this community. This community capacity value was included in the model to limit the exponential growth of the owned dog population. Once the community capacity value was reached in the model, any newborn dogs added to the population were immediately removed from the dog population, until the population size of dogs decreased below the community capacity. The modeled population of dogs was assumed to be open with births and deaths, and immigration and emigration (further described below). Individual male and female dogs were characterized by defined life cycle states (stages) described in Fig 1 (males) and Fig 2 (females).

The model considered aging between life cycle states. The model was run for a period of 20 years, with one time-step representing one year.

### 2.3. Model parameterization

The model was parameterized using a combination of empirical data for Villa de Tezontepec, Hidalgo, Mexico [13], wherever possible, and data available from the peer-reviewed literature where empirical data specific to this case study were not available. A complete description of all model parameters is provided in Table 1.

**Table 1:** Model parameters describing the transition rates and/or times for individual dogs to move between the different model states. Single fixed values are indicated by “N/A” in the distribution column.

Parameter	Value	Distribution (distribution parameters)	Reference
**General parameters**			
Time to weaning	8 weeks	N/A	[22]
Time to sexual maturity female	Average 6 to 10 months	Uniform (Min. 6 months Max. 10 months)	[22]
Time to sexual maturity male	Average 10 months	N/A	[22]
Puppy annual risk of non-age-related mortality confined	0.10 per year (Sensitivity analysis range from 0.05 to 0.30)	N/A	Tables A-E in S1 File (Sensitivity analysis: [23–26])
Puppy annual risk of non-age-related mortality unconfined	0.20 per year	N/A	Assumption (2 X Puppy annual risk of non-age-related mortality confined) (Sensitivity analysis: based on researchers’ hypothesis)
Young annual risk of non-age-related mortality confined	0.20 per year (Sensitivity analysis range from 0.10 to 0.30)	N/A	Tables A–E in S1 File (Sensitivity analysis: [23–26])
Young annual risk of non-age-related mortality unconfined	0.40 per year	N/A	Assumption (2 X Young annual risk of non-age-related mortality confined) (Sensitivity analysis: based on researchers’ hypothesis)
Adult annual risk of non-age-related mortality confined	0.03 per year (Sensitivity analysis range from 0.015 to 0.075)	N/A	Tables A–E in S1 File (Sensitivity analysis: [23–26])
Adult annual risk of non-age-related mortality unconfined	0.06 per year	N/A	Assumption (2 X Adult annual risk of non-age-related mortality confined) (Sensitivity analysis: based on researchers’ hypothesis)
Sterilized risk of non-age-related mortality confined	0.027 per year	N/A	Assumption (90.00% of Adult annual risk of non-age-related mortality confined) (Sensitivity analysis: based on researchers’ hypothesis)
Sterilized risk of non-age-related mortality unconfined	0.054 per year	N/A	Assumption (2 X Sterilized risk of non-age-related mortality confined) (Sensitivity analysis: based on researchers’ hypothesis)
Male risk of age related mortality		Exponential (Min. 0.08 years, Max. 14.00 years, Skewness 2.27 and Kurtosis 10.24)	Tables A–E in S1 File
Female risk of age related mortality		Exponential (Min. 0.50 years, Max. 12.00 years, Skewness 1.58 and Kurtosis 5.13)	Tables A–E in S1 File
Sterilized male risk of age related mortality		Exponential (Min. 0.88 years, Max. 15.40 years, Skewness 2.27 and Kurtosis 10.24)	Assumption (90.00% of Male age-related mortality) (Sensitivity analysis: based on researchers’ hypothesis)
Sterilized female risk of age related mortality		Exponential (Min. 0.55 years, Max. 13.20 years, Skewness 1.58 and Kurtosis 5.13)	Assumption (90.00% Female age-related mortality) (Sensitivity analysis: based on researchers’ hypothesis)
**Female dog only parameters**			
Duration of heat	18 days (Proestrus Average 9 days + Estrus Average 9 days)	N/A	[22]
Gestation duration	65 days	N/A	[27]
Time to New proestrus	Average 7 months	N/A	[22]
Litter size	4 puppies	N/A	[13]
Risk of pregnancy confined	0.26 per year (Sensitivity analysis range from 0.10 to 0.40)	N/A	[13] (Sensitivity analysis: based on researchers’ hypothesis)
Risk of pregnancy unconfined	0.52 per year	N/A	Assumption (2 X risk of pregnancy confined) (Sensitivity analysis: based on researchers’ hypothesis)
**Population parameters**			
Annual probability of immigration	0.23 per year	N/A	Tables A–E in S1 File
Annual probability of emigration	0.04 per year	N/A	Tables A–E in S1 File
Community capacity	2924 dogs	N/A	[13]

Age related mortality parameters (representing the risk of mortality due to old age) and non-age-related mortality parameters (representing the risk of mortality due to traffic accidents, disease, etc.), were considered in the model (Table 1). Non-age-related risk of mortality did not differ between males and females but did differ between confined and unconfined dogs. It was assumed that unconfined dogs were exposed to more risk when free-roaming and as a result their risk of mortality due to non-age-related causes was higher than for confined dogs. Age related mortality was different for males and females, based on empirical data collected in Villa de Tezontepec, Hidalgo, Mexico (Table 1). Model parameters included both static, fixed parameter values, and parameter distributions for model parameters for which there was sufficient empirical data from Villa de Tezontepec, Hidalgo, Mexico (Table 1). Initial model conditions were based on empirical data [13] (Table 2).

**Table 2:** Initial conditions of the agent-based model evaluating owned dog population control interventions in Villa de Tezontepec, Hidalgo Mexico.

Parameter	Values	Reference
**Population parameters**		
Proportion of confined dogs	45.00%	[13]
**Female dogs**		
Population size	1222 dogs	[13]
Proportion puppy	4.00%	Tables A–E in S1 File
Proportion young	11.00%	Tables A–E in S1 File
Proportion in heat (reproductive)	21.00%	Tables A–E in S1 File
Proportion pregnant	6.00%	Tables A–E in S1 File
Proportion not in heat	21.00%	Tables A–E in S1 File
Proportion regular spayed	37.00%	[13]
**Male dogs**		
Population size	1702 dogs	[13]
Proportion puppy	6.00%	Tables A–E in S1 File
Proportion young	16.00%	Tables A–E in S1 File
Proportion reproductive	64.00%	Tables A–E in S1 File
Proportion regular neutered	14.00%	[13]

For this model, we used the reported proportion of owned dogs kept confined (45%) in Villa de Tezontepec (Table 2). The parameter describing the time to sexual maturity (representing the time that it takes for a female dog to reach sexual maturity and enter her first heat cycle) was described by a uniform distribution (Table 1). A uniform distribution was selected to represent the range of values described in the peer-reviewed literature for this reproductive process [22]. We assumed that female dogs had an equal chance to become reproductively active within the specified range used for the time to sexual maturity for female dogs. Age related mortality was described by an exponential distribution (Table 1). An exponential distribution was selected for these mortality parameters because it was assumed that dogs over a year-old die continuously and independently from other dogs at a constant average rate.

### 2.4. Model parameter assumptions

The model assumed that the community capacity remained constant over the duration of the simulation period (20 years). In the hypothetical population, all female dogs were assumed to be equally fertile. Our assumption therefore provided a worst-case scenario in terms of population size. The model also assumed that the risk of pregnancy was different for confined and unconfined female dogs. Unconfined female dogs were assumed to have a higher risk of pregnancy as described by the empirical data for Villa de Tezontepec, Hidalgo, Mexico (Table 1, Tables A–E in S1 File). Unconfined dogs were also assumed to have a higher risk of mortality [28]. For this model, we assumed that the risk of mortality for unconfined dogs was double the value of the risk of mortality of confined dogs (Table 1). It has been reported that sterilization increases a dog’s life expectancy [29]. Therefore, we assumed that both non-age-related mortality and age-related mortality were different for dogs that were sterilized compared to those that were not (Table 1). For this model, it was assumed that sterilization increased annual survival by 10% [29]. This assumption was based on findings reported by Hoffman et al. (2013) [29], which indicated that sterilization increases life expectancy by 13.8% in males and 26.3% in female dogs. These reported results are based on data from North American veterinary teaching hospitals, which might not reflect the health condition of animals in Villa de Tezontepec. As a result, we constrained our assumption to a conservative 10% increase in annual survival for sterilized owned dogs.

### 2.5 Reproduction

Reproduction is determined by the risk of pregnancy for female dogs and the availability of intact animals in the hypothetical population. In the model, female dogs will continue to reproduce provided that there is at least one intact male in the hypothetical population.

### 2.6. Immigration and emigration

Immigration and emigration events occurred four times a year (i.e. every 0.25 years) in the model. At each immigration event, both female and male dogs were randomly added to the population in the “Puppy” state. Empirical data for Villa de Tezontepec, Hidalgo, Mexico (Table C in S1 File) indicated that the majority of owned dogs that immigrate to the area do so as puppies, and that these animals are purchased, adopted, or received as gifts from nearby municipalities. As such, each of the dogs added to the population as part of an immigration event were assumed to be intact and non-reproductive. At each emigration event, a proportion of female and male dogs were randomly removed from the population across all age groups and reproductive statuses. For our model, we assumed that during an emigration event, dogs (of any age) were sold or given away by their owners to families or humane societies located outside the population area, because they could no longer take care of them.

### 2.7 Model life cycle states

#### 2.7.1. Male dogs

Male dogs followed the life cycle states described in Fig 1. This cycle included age-specific states labeled Puppy (aged birth to 8 weeks), Young (aged 8 weeks to 8 months), and Adult (8 months and older). The choices for differentiation between age-specific states relate to modeling the impacts of population control interventions, rather than biological age. Transitions (arrows) governing the movement of male dogs from one state to another (Fig 1) are described in Table 1. In the model, when male dogs grow into adults, they transition immediately to the Reproductive state (indicating that they have reached sexual maturity and are able to reproduce). A separate Neutered state was included in the model to indicate male dogs that had been surgically sterilized (Fig 1).

#### 2.7.2. Female dogs

The life cycle of female dogs in the model is described in Fig 2. Female dogs could be in one of three mutually exclusive age-based states that included Puppy (aged birth to 8 weeks), Young (aged 8 weeks to 4–8 months), and Adult (4–8 months and older, depending when the dog reached its sexual maturity in the model). The Adult state included three mutually exclusive sub-states to describe the female reproductive cycle: In Heat (Reproductive), Pregnant, and Not in Heat. An independent Spayed state was also included in the model to designate female dogs that had been surgically sterilized (Fig 2). Transitions (arrows) governing the movement of female dogs from one state to another (Fig 2) are described in Table 1.

When female dogs became adults in the model, they transitioned immediately to the In Heat state (representing their first heat event). Dogs in the In Heat state were considered fertile and available for possible mating and subsequent pregnancy. From the Pregnant state, after the gestation period [27] (Fig 2, Table 1), a litter of four new puppies (two male and two female, based on empirical data for the region [13]), were added to the dog population in the Puppy state (Table 1). We assumed that female dogs were only eligible for surgical sterilization if they were between heats. Although government subsidized surgical sterilization programs in the municipality of Villa de Tezontepec might sterilize female dogs in heat or in the early stages of pregnancy, this is a rare occurrence (Health Services in the State of Hidalgo (SSEH), 2016, personal communication); therefore, we simplified the assumption to only consider females between heats as eligible for the surgical intervention.

### 2.8. Model output

Each model simulation scenario used a 20-year time horizon. The primary outcome of interest (final population size) was aggregated across 1000 model iterations by calculating the mean and median population size, standard deviation, and absolute range (minimum to maximum) for each intervention scenario. Standard box plots were used to graph the owned dog population size results. Each set of model outcomes (1000 replicates per intervention) were compared to the base case scenario (i.e. a simulation using the initial model parameter values but in the absence of any interventions), and to each other for each type of intervention (Table 3).

**Table 3:** Model outcomes for the surgical interventions examined using the individual-based model describing dog population dynamics in Villa de Tezontepec, Hidalgo Mexico. For each intervention, the model was run 1000 times with the outcome of interest being the total dog population size after 20 years. Outcomes are aggregated across all model iterations and summarized as mean population size, standard deviation, median population size, range, and relative change compared to the no intervention scenario.

Intervention	Intervention number	Surgical capacity	Mean population size (# of dogs)	Standard deviation	Median population size (# of dogs)	Range (min–max)	% relative change compare to base case
Base case	N/A	N/A	2934	6.20	2936	2878–2945	0.00%
**Surgical sterilization**							
A. Mixed age surgical sterilization	A.1	Level 1–21 surgeries per month	2519	496.71	2881	1443–2937	-14.14%
	A.2	Level 2–42 surgeries per month	1564	341.09	1525	798–2705	-46.69%
	A.3	Level 3–84 surgeries per month	624	91.13	612	418–983	-78.73%
B. Young age surgical sterilization	B.1	Level 1–21 surgeries per month	558	122.06	532	331–1125	-80.98%
	B.2	Level 2–42 surgeries per month	339	29.81	337	261–437	-88.44%
	B.3	Level 3–84 surgeries per month	303	23.72	302	230–402	-89.67%
C. Female only mixed age surgical sterilization	C.1	Level 1–21 surgeries per month	532	55.29	526	392–714	-81.87%
	C.2	Level 2–42 surgeries per month	345	34.37	343	251–513	-88.24%
	C.3	Level 3–84 surgeries per month	235	19.82	233	180–301	-91.99%
D. Female only young age surgical sterilization (prior to sexual maturity)	D.1	Level 1–21 surgeries per month	307	34.71	303	217–427	-89.54%
	D.2	Level 2–42 surgeries per month	287	31.86	285	200–413	-90.22%
	D.3	Level 3–84 surgeries per month	276	28.96	275	190–384	-90.59%

Model outputs were visualized and analyzed using the statistical software package STATA/SE for Mac (StataCorp LP, 2015).

### 2.9. Base case scenario

The model base case scenario used the initial conditions, and model parameter values described, but in the absence of any interventions (Tables 1 and 2).

### 2.10. Surgical sterilization interventions

Surgical sterilization interventions were simulated using our base case model (Table 3). All the surgical sterilization interventions evaluated used the current government sterilization sex targeting ratios described below, and then were scaled up accordingly based on surgical capacity.

We examined four different surgical sterilization intervention strategies. These strategies varied in that they targeted dogs of different sexes (both male and female dogs or female dogs only), dogs of different ages (both sexually mature and sexually immature dogs or sexually immature dogs only), and at different levels of surgical capacity (number of surgeries performed per unit time). The current regional government’s “mixed” approach (sterilization of both female and male dogs, including young, sexually immature dogs and adult dogs) is one of the strategies considered in our model. We modeled a mixed age surgical sterilization intervention scenario and a female-only mixed age surgical sterilization intervention scenario both at three different levels of surgical capacity. Capacity level 1 is based on the number of monthly surgeries (21) currently performed by the Ministry of Health in the State of Hidalgo in Villa de Tezontepec (8.6% annually); on average, this includes 6 adult males, 13 adult females, 1 young male, and 1 young female dog (SSEH, personal communication, 2016). For comparison, we also modeled increased surgical capacity at 42 surgeries per month and 84 surgeries per month using the same sex and age distribution as the lowest surgical capacity described above. The targeted sex and age ratios were adjusted accordingly for the interventions scenarios aimed at female dogs only (Table 3).

There is evidence that targeting surgical sterilization to young female dogs (before they reach sexual maturity) improves dog population control interventions [14]. Therefore, we also examined the potential impact of focusing surgical sterilization interventions on young, sexually immature dogs (female dogs aged 8 weeks to 4–8 months and male dogs aged 8 weeks to 8 months). For the sterilization interventions focused on young animals of both sexes, the sex distribution was adjusted according to the current government surgical sterilization sex distribution. And for the young dog, female-only sterilization interventions, all available surgeries per surgical capacity were focused on sexually immature female dogs only. For these young age surgical sterilization intervention scenarios, we examined the same three levels of surgical capacity as for the mixed age surgical sterilization interventions (21, 42, and 84 surgeries per month) (8.6%, 17.2%, and 34.5% annually)

### 2.11. Sensitivity analyses

We conducted sensitivity analyses to evaluate the impact of parameter uncertainty. The parameter values describing the risk of mortality (non-age related, age related, and the death of sterilized dogs), and the risk of pregnancy for female dogs, both for confined and unconfined dogs, were identified as being of specific concern as they were based on assumptions (empirical data from the region were not available for unconfined dogs). To identify biologically realistic ranges for the sensitivity analyses around the empirical estimates, a literature search for dog demographic studies conducted in Latin America that reported values for the parameters of interest was conducted. The maximum values of the ranges used in the sensitivity analyses (for the mortality parameters), were estimated by calculating the mean value from all the parameters found in the peer-reviewed literature [23–26], for each of the parameters of interest. These values were used as the maximum value of the ranges examined, because the empirical values from Vila de Tezontepec were lower than the calculated mean values obtained from the peer-reviewed literature. The minimum value of the ranges used in the sensitivity analyses, were assumed to be half of the empirical values from Villa de Tezontepec. Since there were no data available from the peer-reviewed literature (in Latin America) for the risk of pregnancy values (the annual probability of becoming pregnant), the lower and upper range values for this parameter were assumed to be plus 14% and minus 16% of the empirical risk of pregnancy from Villa de Tezontepec. These values were selected for simplicity to round the lower and upper range values from 10% to 40%. The range of parameter values used for unconfined dogs in our sensitivity analysis included values between the values used for confined dogs and the assumptions made for unconfined dogs in the model (Table 1). For example, for the annual risk of non-age-related mortality for puppies for unconfined dogs, the analysis included values between (5% and 50%). To test the sensitivity of the risk of mortality for sterilized dogs, we first evaluated the assumption for confined dogs (90% risk of non-age-related mortality). During this evaluation, the risk of mortality for sterilized unconfined dogs was kept as described in Table 1. When the risk of mortality for unconfined sterilized dogs was evaluated, the risk of mortality for confined dogs was kept constant (90% risk of non-age-related mortality), then values of the risk of sterilized mortality for unconfined dogs was varied. For the risk of age related mortality after sterilization, parameters values for male and female dogs were varied simultaneously in the same magnitude within the specified range.

The baseline community capacity for owned dogs in the community was based on the assumption that the current population of owned dogs was at a steady state and that only dog-owning households (as identified in an earlier study [13]), would continue to own dogs. However, it is possible that non-dog-owning households could also be interested in owning dogs if these become available from the owned dog population. Choices related to dog ownership are influenced by human behaviour and other associated factors and these factors may play a more important role that density dependent factors [30]. For owned dog populations, the population size is entirely determined by the choices of humans. To examine the potential impact of our baseline community capacity, we decided to also evaluate an “upper bound” for the community capacity parameter. In this “upper bound” scenario, we assumed all households in the community could potentially own dogs. The “upper bound” community capacity value was calculated by multiplying the total number of households in the municipality (2249 households) [20], by the mean number of owned dogs per household (2 dogs) that has been previously documented in this community [13]. This resulted in an alternative scenario where the community capacity was 4498 owned dogs.

In general, for the range of values determined as biologically reasonable for our sensitivity analysis, all model intervention scenarios were varied independently (all parameters remained unchanged except for the one being investigated) and the model was re-run (1000 iterations). Model outputs were compared by calculating the percent change and absolute difference in population size to identify if changing the model inputs resulted in numerically different model outputs in terms of average projected population size after 20 years.

We present the results of our sensitivity analysis for the different sterilization interventions at the lowest surgical capacity, in standard box plots showing 1000 iterations of each scenario at each variation of sensitivity in the supporting information. Model outputs across the range of parameter values examined were compared and graphed using the statistical software package STATA/SE for Mac (StataCorp LP, 2015).

## 3. Results

### 3.1. Base case simulations

The base case model resulted in a dog population with a mean population size of 2934 dogs (SD = 6.2; median: 2936, range: 2878 to 2945), over 1000 simulations with the maximum dog population size constrained by the pre-defined community capacity of the population (2924 dogs) (Table 3).

### 3.2. Mixed age surgical sterilization intervention

**Fig 3:**
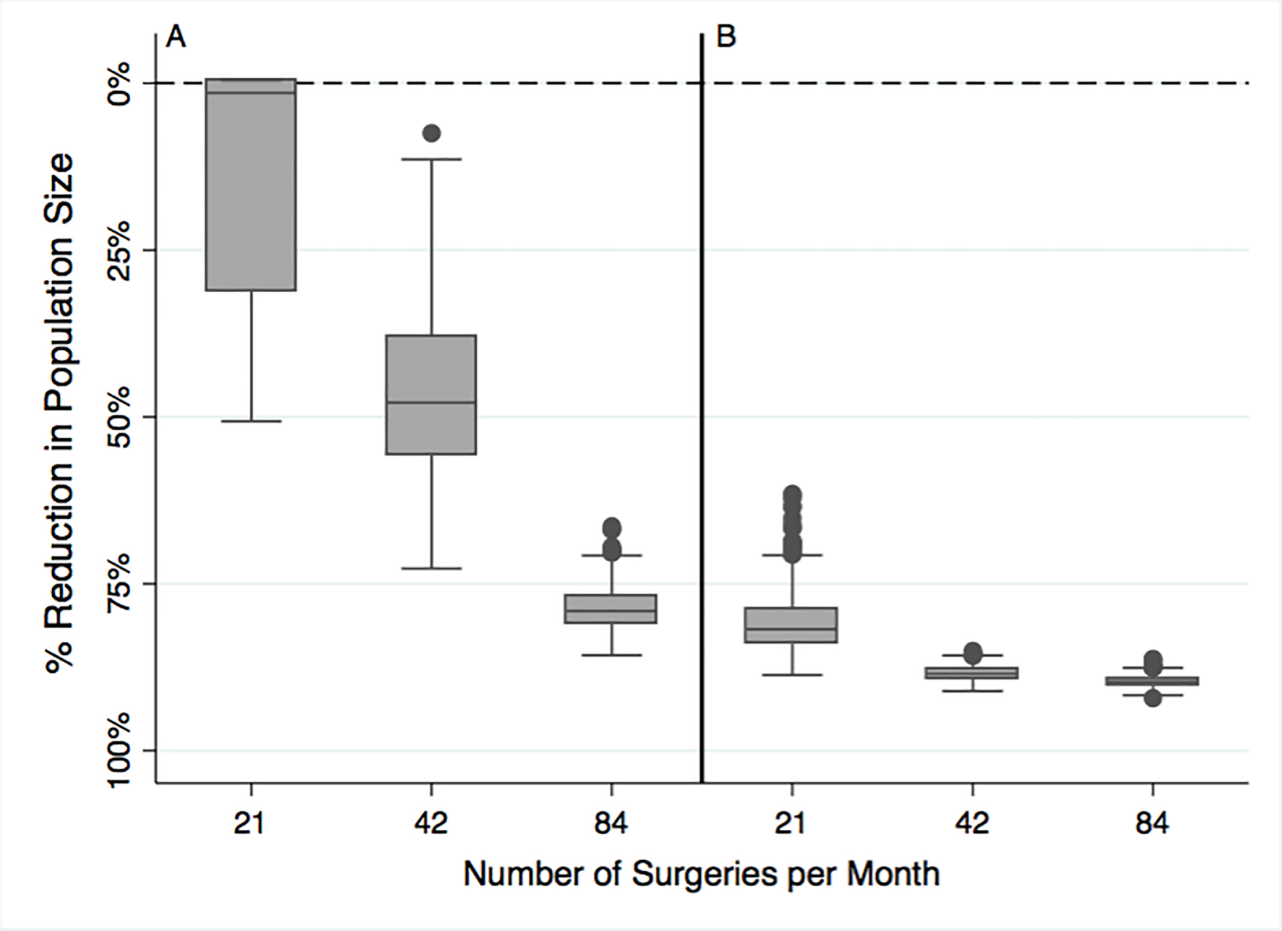
Impact of mixed age (Panel A) and young age (Panel B) surgical sterilization interventions (both male and female dogs) after 20 years. Each box represents a summary of the model outcome (population size) across 1000 stochastic model replicates. The top and bottom of each box are the 25^th^ and 75^th^ percentiles, and the line inside the box is the median dog population size. The top whiskers are the minimum and maximum values of population size, excluding outliers, which are represented in the figure by solid circles. The dashed line represents the population community capacity (2924 dogs).

Using a mixed age surgical sterilization intervention, the median dog population size decreased over the 20-year time period (Fig 3, Table 3).

Mixed age surgical sterilization interventions at the lowest surgical capacity (21 surgeries, 8.6% annually), resulted in a 14.1% reduction in the mean dog population size compared to the base case scenario with no sterilization interventions (Table 3). Intervention scenarios with increased surgical capacity (42 and 84 surgeries per month) had a more pronounced reduction (46.7% and 78.7%) in the dog population size (Fig 3, Table 3).

### 3.3. Young age surgical sterilization intervention

A surgical intervention that used the same surgical capacity (21–84 surgeries per month, 17.2%, and 34.5% annually) but focused the surgical capacity on young, sexually immature dogs resulted in model outcomes where the mean dog population size was markedly reduced, compared to the base case scenario (Table 3). For sterilization interventions that were focused on young sexually immature dogs, as the surgical capacity increased, the median dog population size also decreased (Fig 3). For example, the young age surgical sterilization intervention at the lowest surgical capacity, resulted in a mean dog population size of 558 dogs after 20 years, which represented a mean population size reduction of 81.0% compared to the base case scenario (Table 3). Furthermore, when young age surgical sterilization interventions were compared to the mixed age surgical sterilization interventions with the same level of surgical capacity, the projected percent reduction in population size across the three levels of surgical capacity (21, 42 and 84 surgeries per month were -78.0%, -78.0% and -51.0% (Table A in S2 File). In general, across all levels of surgical capacity, mean population size reductions were greater for surgical interventions focused on young dogs rather than dogs of mixed ages (Fig 3).

### 3.4. Female-only, mixed age surgical sterilization intervention

A surgical intervention targeted at only female dogs (mixed ages) at the lowest surgical capacity (8.6% annually) resulted in an 81.9% reduction in the mean dog population size compared to the base case scenario (Table 3). When the surgical capacity was increased to 42 surgeries (17.2% annually), the projected population size was reduced 88.4% compared to the base case scenario (Table 3). At the highest surgical capacity, reductions increased to 92.0% (Table 3). When the female, mixed age sterilization intervention was compared to the sterilization approach targeting dogs of both sexes with mixed ages, at the lowest surgical capacity the difference in population size was 1987 fewer dogs, corresponding to a 79% population size reduction (Table B in S2 File). As surgical capacity increased, the population size reductions became larger (Table B in S2 File).

### 3.5. Female-only young age surgical sterilization intervention

**Fig 4:**
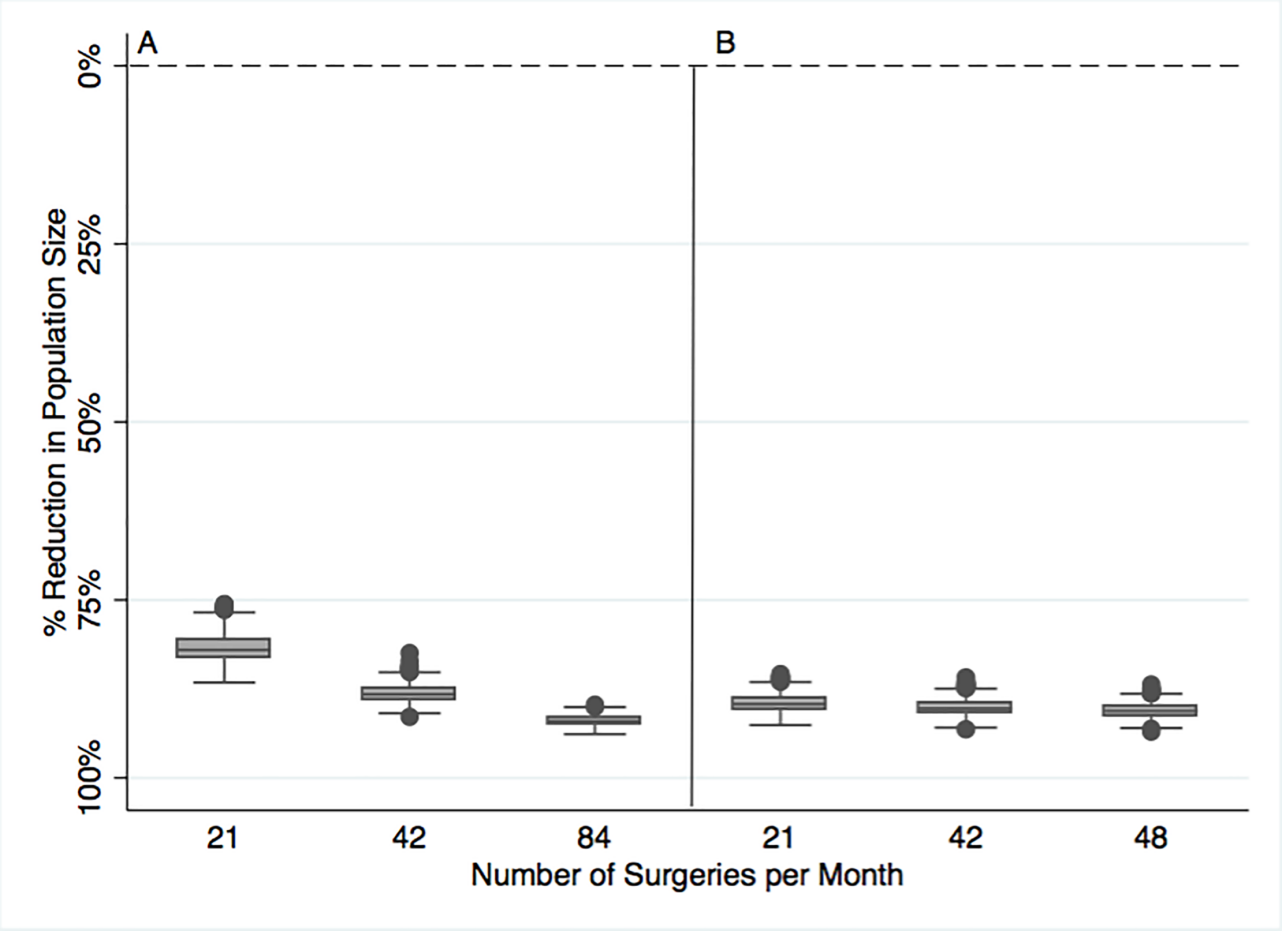
Impact of female only mixed age surgical sterilization (Panel A) and female only young age surgical sterilization (Panel B) interventions after 20 years. Each box represents a summary of the model outcome (population size) across 1000 model replicates. The top and bottom of each box are the 25^th^ and 75^th^ percentiles, and the line inside the box is the median dog population size. The top whiskers are the minimum and maximum values of population size, excluding outliers, which are represented in the figure by solid circles. The dashed line represents the population community capacity (2924 dogs).

Surgical interventions focused on only sexually immature female dogs, resulted in even greater population size reductions compared to the base case scenario (Table 3). At the lowest surgical capacity, the model projected an 89.5% reduction in population size for this intervention (Table 3). When surgical interventions treating exclusively young female dogs were compared to surgical interventions open to female dogs of any age, no considerable difference in population size reduction was observed (Fig 4).

The population size difference between these two interventions was more pronounced when both interventions were compared at the lowest level of surgical capacity (Table C in S2 File). Surgical interventions directed towards young female dogs resulted in slightly larger population size reduction compared to surgical interventions directed towards female dogs of any age (Table 3). When surgical interventions aimed solely at young female dogs were compared to surgical interventions accessible to young dogs of mixed sexes, a projected 45% population size reduction was observed for the lowest level of surgical capacity. However, no considerable population size reduction was observed for higher level of surgical capacity (Table D in S2 File).

### 3.6. Sensitivity analyses

For confined dogs, varying the annual risk of non-age-related mortality for adult dogs from 1.5–7.5% had a more pronounced effect as the risk of mortality increased (Figure A in S3 File). Varying the annual risk of non-age-related mortality for puppies (from 5% to 30%) and young dogs (from 10% to 30%) led to greater variability in the projected model outcomes (Figures B and C in S3 File). For unconfined dogs, when the risk of non-age-related mortality dogs increased for young from 10% to 60%, the mean population size decreased and the model outcomes were more variable. (Figure D in S3 File). Varying the annual risk of non-age-related mortality for puppies (from 5% to 50%) and adult dogs (from 1.5% to 9%) resulted in outcomes with little variability (Figures E and F in S3 File). A decrease in the risk of pregnancy for unconfined dogs between 66% and 10% led to a considerable population size reduction, in addition, as the risk of pregnancy decreased the model projections became less variable (Figure G in S3 File). Similarly, a reduction in the risk of pregnancy for confined dogs (from 26% to 10%) resulted in a less variable and more attractive outcome (Figure H in S3 File).

An increase in the community capacity parameter value from 2924 (baseline community capacity) to 4498 dogs (full community capacity), on the outcomes for the mixed age and young female only, surgical sterilization interventions at the lowest surgical capacity (21 surgeries, 8.6% annually), led to almost no population size reduction for the mixed age intervention under the higher community capacity assumption (Figure I in S3 File). However, when the increase in community capacity was evaluated for the young female sterilization intervention also at the lowest surgical capacity, a considerable population size reduction was observed (Figure I in S3 File). For both levels of the community capacity considered, the surgical intervention focused on young female dogs demonstrated more significant reductions in the overall population size compared to the mixed age intervention. These sensitivity analyses focused on the community capacity assumption provide support for the finding that surgical sterilization focused on young, female dogs has a greater impact then mixed aged sterilization in both scenarios.

In general, model outcomes were more sensitive to the risk of pregnancy and non-age-related mortality for both confined and unconfined dogs which influenced the relative effectiveness of the interventions evaluated. An increase in the community capacity influenced the effectiveness of the intervention that targeted dogs of mixed age and sexes but did not change the relative effect when the intervention focused on young female dogs only (Figures I and J in S3 File). Variation in the mortality risk of sterilized dogs did not affect model outcomes (Figures K–L in S3 File).

## 4. Discussion

The control of dog reproduction is one of several measures that can be used to control the size of dog populations [3]. Surgical sterilization is the most commonly used method of pet contraception [4]. Several mathematical models have been developed to evaluate the effect of sterilization programs for both owned and free-roaming dog populations in different parts of the world, including Italy [14], Brazil [15–17], and India [18–19]. No models have been developed to evaluate the effect of ongoing surgical sterilization programs aimed at owned dogs in Mexico. Currently, the government sterilization program in Villa de Tezontepec has a maximum surgical capacity of 21 surgeries per month (equivalent to the sterilization of 8.6% of the current owned dog population per year) and focuses on dogs of mixed ages and sexes. Our model suggests that the long-term deployment of the mixed age surgical sterilization intervention at the lowest surgical capacity examined (in line with the existing program), combined with the current proportion of owned dogs that are confined (45%), and an initial population of sterilized dogs (37% females and 14% males), would only reduce the mean dog population by approximately 14.4% after 20 years. Our baseline model projections are comparable to those reported by Baquero et al. (2016) [15], where an annual probability of sterilization of 12% and 8% for females and males resulted in a 17% reduction in the owned dog population reduction after 30 years. We used our model to examine the impact of modifying the existing surgical capacity, as well as the ages and sexes of the dogs receiving the intervention. Our findings suggest that if the number of sterilizations were to double (42 surgeries/month: 18.7% of the owned dog population sterilized annually), the owned dog population could decrease by 46.7% in the same 20-year time period. However, such surgical increases may be difficult to sustain over the long term for a single region, due to the costs and required program resources.

Our model projections suggest that surgical sterilization interventions directed exclusively at female dogs of mixed ages could be more effective at reducing the dog population than interventions open to dogs of any age and sex in the long term (20 years). Our results demonstrate a mean population reduction of 81.9% and 88.4% at surgical capacities of 21 and 42 surgeries per month (8.6% and 17% of the owned population sterilized annually). Amaku et al. (2010) reported that for a stray dog population, a population size reduction of 50% could be achieved in less than 10 years if the probability of sterilization for female dogs was higher than 20% of the total dog population being sterilized per year [17]. It is important to note that the model proposed by Amaku et al. (2010) [17] described the effect of sterilization in a stray dog population. The population dynamics (i.e. birth and mortality rates, immigration and emigration) of stray dogs can be very different from owned dogs. This is primarily due to human behaviour which can influence the population dynamics of dogs (stray or owned) and act to increase or decrease their abundance [31–32].

Our model suggests that surgical sterilization interventions focused only on female dogs could improve the current government dog population control efforts in this location, without investing extra resources. Previous studies suggest that dog population control programs should focus on surgical sterilization of young female dogs (i.e. before they have a first litter) in order to be most effective [14]. Our results provide further support for this assertion. In our model, the most significant population size reduction (90%) was observed when surgical sterilization at the current government surgical capacity (21 dogs per month, 8.6% of the owned dog population per year), was applied to only female, sexually immature dogs. Qualitatively, our findings are in line with those described by Di Nardo et al. (2007). Using a model to describe the population dynamics of the owned dog population in an Italian province, the authors have demonstrated that within the Italian context, surgical sterilization interventions focused on young, female dogs have a more significant impact on long term population dynamics than interventions focused on female dogs from all age groups [14]. However, in the Mexican context, we find that adding increased surgical capacity (84 surgeries per month) to the young female sterilization intervention did not yield further population size reductions but rather, at the highest level of surgical intervention, focusing on female dogs of all ages resulted in greater reductions in the final population size. We hypothesize that this outcome is likely the result of having a very limited number of young female dogs left to sterilize as time passes. Therefore, sterilizing only young female dogs at high surgical capacities does not improve the effect of the surgical sterilization intervention because at a certain point, there are very few young female dogs remaining in the population. For this reason, as the dog reproduction control program continues, there will be a need to change the focus to other age-sex intact classes for the program to remain effective. Di Nardo et al. have also suggested that their model is highly sensitive to the mortality rate of the dogs in the model. Since we had access to survey data that described the proportion of owned dogs that were allowed to roam free in this community (55%) [13], we assumed that these animals had a higher risk of mortality than confined dogs. This elevated mortality rate for a large number of the owned dogs in the model, could have led to a larger population size reduction overtime. Baquero et al. (2016) suggest that the effect of sterilization interventions should be evaluated on the variation of the infertile population fraction rather than on the population [15]. In general, it appears that surgical sterilization efforts focused at young female dogs could enhance the efficacy of the current surgical intervention for the owned dog population in this community, without the need to increase the number of sterilization surgeries performed per month.

Model sensitivity analyses were performed to evaluate the possible impact of changing assumptions regarding the model parameters for pregnancy, mortality for both confined and unconfined dogs, and community carrying capacity. Changes to the risk of non-age-related mortality (within reasonable limits, based on a review of the literature) increased the variability of the model results, and the attractiveness of surgical interventions in terms of relative benefit. Our sensitivity analysis also indicated that for confined dogs, an increase in the risk of non-age related mortality in young and adult dogs had a greater effect on the model outcomes than an increase in the risk of mortality in puppies. However, for unconfined dogs, only the increase in the risk of non-age-related mortality for young dogs had a considerable effect on the model outcomes. Our sensitivity analysis also examined model outcomes across a range of pregnancy values for confined and unconfined dogs and draw attention to the finding that surgical sterilization interventions become more attractive as the baseline risk of pregnancy is reduced. This result suggests that by reducing the interest and/or willingness of owners to breed their pets, the interventions examined here are expected to have an even greater impact. Carrying capacity is one of the most influential parameters in dog population model simulations [15]. Model sensitivity analyses for the community capacity parameter suggest that as the population size increases, the effectiveness of interventions decreases for interventions focused on dogs of any age and sex. However, when we examined the community capacity on interventions targeting exclusively sexually immature female dogs, our model projections were robust to the changes in community capacity assumptions. This finding suggests that surgical sterilization interventions aimed at young female dogs could continue to be effective over time even if the size of the owned dog population were to increase (e.g. due to human behaviour or dog immigration). In general, uncertainty around the baseline risk of pregnancy and non-age-related mortality parameters, make the model relatively sensitive to these assumptions. As a result, the projected impact of the modeled interventions demonstrates increased variability as these parameters increase across a range of biologically realistic values. This highlights the need for robust estimates of dog population numbers and attributes, including risk of mortality and pregnancy estimates, especially when models are used to evaluate population control interventions [14]. Improved data availability for these specific parameter values would allow for the refinement of the model projections.

## 5. Limitations

All models are simplifications of reality and therefore, as with any model-based analysis, ours has limitations. This model only considered the owned dog population in this region and did not consider stray (non-owned, free-roaming dogs) or feral (dogs living in a “wild and free state” without intentional or direct food or shelter provided by people) dog populations [33]. Therefore, this model only describes population dynamics for a subset of all dogs within this community. Consequently, the results of these model simulations do not reflect the total impact of the interventions on the overall dog population size for this community. Current surgical sterilization interventions in this region are exclusively targeted at owned dogs and the outcome of this model can help inform current programs for the owned dog population in this region. For this reason, we believe it was appropriate to model only the owned dog population to evaluate the effect of surgical sterilization interventions in this subset of the dog population. For this model, we assumed that all dogs that were unconfined (completely or partially allowed to roam), within their age group, had equal risk of non-age-related mortality, and that all unconfined female dogs had the same pregnancy risk; however, in reality, dogs allowed to roam all day might have higher risk of mortality and pregnancy than those that are allowed to roam only part of the day. In addition, it was assumed that all dogs that immigrated to the hypothetical population did so as puppies (less than 8 weeks old). Even though empirical data from this region suggests that this was a reasonable assumption, this may have influenced the effectiveness of interventions being studied. This model did not consider the growth of the human population in the 20-year period, which could influence the community capacity used for this model. One of the implications of capping the owned dog population size in the model (i.e. establishing a community capacity) was not being able to detect the growth of the owned dog population over time beyond the community capacity, both in the absence and presence of dog population control interventions (i.e. surgical sterilization control). Another limitation of this model is that it was not spatially specific; therefore, we do not know if the effect of the interventions varies by neighbourhood. We also assumed that participation in the sterilization intervention was equally distributed across region. In reality, heterogeneous socioeconomic levels could impact the participation of dogs in the subsidized sterilization program and the enrolment might not be evenly distributed in Villa de Tezontepec. Lastly, another considerable limitation of our model is that it did not take in account the owners’ decision making, such as the willingness to breed their dogs or to participate in dog population control initiatives. Owned dog populations and their management are highly influenced by owner decision making. For example, dog owners that breed their dogs for profit would be less likely to participate dog reproduction control interventions; this was not considered in the model. This model could be expanded to incorporate the free-roaming, non-owned dog population to evaluate population control methods for the total dog population in this location, if appropriate data resources were to become available.

## 6. Conclusion

Controlling the growth of the owned dog population is very important for the sustainability of the federally subsidized rabies vaccination program in Mexico [10]. Our model findings suggest that the sterilization of young, sexually immature dogs, especially females, could be more successful at reducing the owned dog population size over a 20-year time horizon compared to the current strategy of sterilization focused on dogs of any age and sex. Reducing the proportion of owned female dogs becoming pregnant also had a significant effect on the overall owned dog population size, suggesting that if owners were discouraged from breeding their female dogs, this alone could reduce the owned dog population size. Computer simulation models can help governments and other decision-makers explore options for optimizing the limited resources allocated for dog population management programs.

## Supporting information

S1 File Empirical data from Villa de Tezontepec, Hidalgo, Mexico, 2015, used to determine parameter values for the individual-base model. (DOCX)

S2 File Table A. **Difference in mean population size between mixed age surgical sterilization and young age surgical sterilization interventions within the same level of surgical capacity. Level 1 represents a surgical capacity of 21 surgeries per month, Level 2 represents 42 surgeries per month, and Level 3 represents 84 surgeries per month. Percentages in brackets are the % reduction in mean population size between the two interventions.** Table B. **Difference in mean population size between mixed age surgical sterilization and female only mixed age surgical sterilization interventions within the same level of surgical capacity. Level 1 represents a surgical capacity of 21 surgeries per month, Level 2 represents 42 surgeries per month, and Level 3 represents 84 surgeries per month. Percentages in brackets are the % reduction in mean population size between the two interventions.** Table C. **Difference in mean population size between female only mixed age surgical sterilization and female only young age surgical sterilization interventions within the same level of surgical capacity. Level 1 represents a surgical capacity of 21 surgeries per month, level 2 represents 42 surgeries per month, and level 3 represents 84 surgeries per month. Percentages in brackets are the % reduction in mean population size between the two interventions.** Table D. **Difference in mean population size between young age surgical sterilization and female only young age surgical sterilization interventions within the same level of surgical capacity. Level 1 represents a surgical capacity of 21 surgeries per month, level 2 represents 42 surgeries per month, and level 3 represents 84 surgeries per month. Percentages in brackets are the % reduction in mean population size between the two interventions.**
(DOCX)

S3 File Figure A. **Comparison of the impact of increasing the annual risk of non-age related mortality for adult confined dogs from 1.5% to 7.5% for the mixed age sterilization interventions at the lowest level of surgical capacity. Model outcomes demonstrated increased variability as the adult mortality rate increased.** Figure B. **Comparison of the impact of increasing the annual risk of non-age related mortality for puppies confined from 5% to 30% for the mixed age sterilization intervention at surgical capacity of 21 surgeries per month. Model outcomes demonstrated subtle changes in the relative impact of the interventions under a wide range (5% - 30%) of values for this parameter value; however, these changes introduced the greatest variability in outcome for the highest level of puppy mortality**. Figure C. **Projected impact of increasing the annual risk of non-age-related mortality for young confined dogs from 10% to 30% for the mixed age sterilization intervention (surgical capacity = 21 surgeries per month). Model outcomes appear highly sensitive to this parameter value with higher mortality rates associated with significant variability in model outcomes.** Figure D. **Comparison of the impact of increasing the annual risk of non-age-related mortality for young unconfined dogs from 10% to 60% on the outcomes for the mixed age sterilization intervention at the lowest surgical capacity. Increasing the mortality rate for this age group resulted in more variability in the model outcomes.** Figure E. **Comparison of the impact of increasing the annual risk of non-age related mortality for unconfined puppies from 5% to 50% for the mixed age sterilization at the lowest surgical capacity. Model outcomes appear relatively stable across a wide range of this parameter value.** Figure F. **Comparison of the impact of increasing the annual risk of non-age related mortality for adult unconfined dogs from 1.5% to 9% on the outcomes for the mixed age sterilization intervention at the lowest surgical capacity. In general, the model outcome appears relatively robust to variability in this parameter value.** Figure G. **Comparison of the impact of increasing the annual risk of pregnancy of female unconfined dogs from 10% to 66% on the outcomes for the mixed age sterilization intervention at the lowest surgical capacity. Model outcomes were sensitive to this parameter value with increases in the pregnancy rate resulting in a less favourable intervention outcome.** Figure H. **Comparison of the impact of increasing the annual risk of pregnancy of female confined dogs from 10% to 40% for the mixed age sterilization interventions at a surgical capacity of 21 surgeries per month**. **Increasing the pregnancy rate to 0.4 resulted in far less change in the population size as a result of the intervention than at lower pregnancy rates.** Figure I. **Comparison of increasing the community capacity community from 2924 dogs (Panel A–Baseline community capacity) to 4498 dogs (Panel B- Full Community Capacity), on the outcomes of mixed age and young female only surgical sterilization interventions at the lowest surgical capacity (21 surgeries per month). In general, model outcomes appear relatively robust to variability in this parameter value, especially when the intervention focuses on sexually immature female dogs exclusively.** Figure J. **Comparison of the impact of increasing the risk of non-age-related mortality for sterilized confined dogs from 1.5% to 3.6% on the outcomes for the mixed age sterilization intervention at the lowest surgical capacity. In general, model outcomes did not demonstrate considerable changes in the relative impact of the interventions across the range of values examined.** Figure K. **Comparison of the impact of increasing the risk of non-age related mortality for sterilized unconfined dogs from 2.7% to 6.0% on the outcomes for the mixed age sterilization intervention at the lowest surgical capacity. In general, model outcomes did not demonstrate substantial changes in the relative impact of the interventions across the range of values examined.** Figure L. **Comparison of the percentage change in the risk of age related mortality for sterilized dogs from -20% to +20% on the outcomes for the mixed age sterilization intervention at the lowest surgical capacity. In general, model outcomes did not demonstrate drastic changes in the relative impact of the interventions across the range of values examined.**
(DOCX)
