# Supplementary material for: Modeling the effect of surgical sterilization on owned dog population size in Villa de Tezontepec, Hidalgo, Mexico, using an individual-based computer simulation model
Source: PLoS One. 2018 Jun 1;13(6):e0198209. doi: 10.1371/journal.pone.0198209 (PMC5983437; doi:10.1371/journal.pone.0198209)
Supplement: S1 File — (DOCX) [file pone.0198209.s001.docx]

| **Age Group** | **Total # of Owned Dogs**  **# (%)^e^** | **Percent from Total Population^f^** | **Owned Dogs**  **Median Age**  **(range)** | **Owned Dogs Mean Age**  **(SD)** |
| --- | --- | --- | --- | --- |
| Female young | 11 (18.64) | 11.22 (0.19*0.59) | 0.5 (0.21 - 0.75) | 0.50 (SD = 0.19) |
| Female Pregnant | 6 (10.17) | 6.12 (0.10*.059) | 1.75 (0.67 – 6.00) | 2.28 (SD = 1.89) |
| Female Adult^ab^ | 42 (71.19) | 41.13 (0.71*0.59) | 3.00 (1.00 – 11.00) | 3.37 (SD = 2.11) |
| **Total Female^c^** | **59 (100.00)** | **59.20 (1.00*0.59)** | **2.00 (0.21 – 11.00)** | **2.70 (SD = 2.17)** |
| Male young | 23 (19.83) | 15.92 (0.20*0.80) | 0.5 (0.25 - 0.83) | 0.51 (SD = 0.17) |
| Male Adult | 93 (80.17) | 64.39 (0.80*0.80) | 3.00 (0.92 – 15.00) | 3.34 (SD = 2.47) |
| **Total Male^d^** | **116 (100.00)** | **80.30 (1.00*0.80)** | **2.00 (0.25 – 15.00)** | **2.78 (SD = 2.48)** |

**S1 Table. Empirical data from Villa de Tezontepec, Hidalgo, Mexico, 2015, used to determine parameter values for the individual-base model.**

1. **Minimum, maximum, mean, standard deviation and total number of owned dogs in the young, pregnant, and adult age group categories**

a. Includes In heat and Not in heat

b. Excludes female adult pregnant dogs

c. Excludes female puppies and spayed dogs

d. Excludes male puppies and neutered dogs

e. Includes only owned dog data where numerical age values were provided

f. Based on total dog population (n=428). Calculation= Total number of dogs in age group divided by total number of dogs per gender, multiplied by percentage of total population per gender

1. **Minimum, maximum, mean, standard deviation and total number of owned dogs older than one year. Population distribution skewness and kurtosis also included**

| **Age Group** | **Total # of Owned Dogs**  **# (%)** | **Owned Dogs**  **Median Age**  **(range)** | **Owned Dogs Mean Age**  **(SD)** | **Skewness** | **Kurtosis** |
| --- | --- | --- | --- | --- | --- |
| Female > 1 year old | 72 (41.62) | 3.00 (1.5 – 13.00) | 3.87 (SD = 2.56) | 1.59 | 5.14 |
| Males > 1 year old | 101 (58.38) | 3.00 (1.08 – 15.00) | 3.70 (SD = 2.30) | 2.27 | 10.24 |
| **Total** | **173 (100.00)** | **3.00 (1.08 – 15.00)** | **3.77 (SD = 2.41)** | **1.95** | **7.64** |

1. **Percentage and total number of owned dogs that immigrated and emigrate to and from Villa de Tezontepec, Hidalgo Mexico, 2015**

| **Category** | **Total per Category**  **# (%)** | **Percentage from Total Population^a^** |
| --- | --- | --- |
| Number of dogs purchased outside the population | 39 (40.21) | 9.11 (39/428) |
| Number of dogs that were a gift from outside the population | 58 (59.79) | 13.55 (58/428) |
| **Total number of dog that came from outside the population** | **97 (100.00)** | **22.66 (97/428)** |
| Number of dogs given away | 9 (50.00) | 2.10 (9/428) |
| Number of dogs sold away | 9 (50.00) | 2.10 (9/428) |
| **Total number of dog that left the population** | **18 (100.00)** | **4.21 (9/428)** |

a. Calculate based on the total population size (n=428)

1. **Percentage and total number of owned dogs that died in the past 12 months in Villa de Tezontepec, Hidalgo Mexico, in 2015**

| **Age Group** | **Total # of Owned Dogs that Died in the Past 12 Months**  **# (%)** | **Total # of Owned Dogs per Age Group^a^** | **Percentage from Total Population per age group** |
| --- | --- | --- | --- |
| Puppies | 2 (8.70) | 21 | 9.52 (2/21) |
| Young | 13 (56.52) | 80 | 16.25 (13/80) |
| Adult^b^ | 8 (34.78) | 294 | 2.72 (8/294) |
| **Total** | **23 (100.00)** | **395** | **5.82 (23/395)** |

1. Includes only owned dog data where age range values were provided
2. Excludes adult dogs older than 5 years
3. **Percentage and total number of female owned dogs that got pregnant in the past 12 months per level of confinement in Villa de Tezontepec, Hidalgo Mexico, in 2015^a^**

| **Category** | **Total # of Female Owned Dogs that got Pregnant in the Past 12 Months per Category** | **Percentage of Female Owned Dogs per category** |
| --- | --- | --- |
| Always Confined | 13 | 38.24 (13/34) |
| Partially Confined | 21 | 61.76 (21/34) |
| **Total** | **34** | **100.00 (34/34)** |

1. Table does not include 3 missing responses.
2. Partially confined Includes: 1) Never confined 71.43% (15/21), 2) Sometimes confined 23.81% (5/21), 3) Confined only at night 4.76% (1/21).
