# Supplementary material for: Modeling the effect of surgical sterilization on owned dog population size in Villa de Tezontepec, Hidalgo, Mexico, using an individual-based computer simulation model
Source: PLoS One. 2018 Jun 1;13(6):e0198209. doi: 10.1371/journal.pone.0198209 (PMC5983437; doi:10.1371/journal.pone.0198209)
Supplement: S2 File — Table A. Difference in mean population size between mixed age surgical sterilization and young age surgical sterilization interventions within the same level of surgical capacity. Level 1 represents a surgical capacity of 21 surgeries per month, Level 2 represents 42 surgeries per month, and Level 3 represents 84 surgeries per month. Percentages in brackets are the % reduction in mean population size between the two interventions. Table B. Difference in mean population size between mixed age surgical sterilization and female only mixed age surgical sterilization interventions within the same level of surgical capacity. Level 1 represents a surgical capacity of 21 surgeries per month, Level 2 represents 42 surgeries per month, and Level 3 represents 84 surgeries per month. Percentages in brackets are the % reduction in mean population size between the two interventions. Table C. Difference in mean population size between female only mixed age surgical sterilization and female only young age surgical sterilization interventions within the same level of surgical capacity. Level 1 represents a surgical capacity of 21 surgeries per month, level 2 represents 42 surgeries per month, and level 3 represents 84 surgeries per month. Percentages in brackets are the % reduction in mean population size between the two interventions. Table D. Difference in mean population size between young age surgical sterilization and female only young age surgical sterilization interventions within the same level of surgical capacity. Level 1 represents a surgical capacity of 21 surgeries per month, level 2 represents 42 surgeries per month, and level 3 represents 84 surgeries per month. Percentages in brackets are the % reduction in mean population size between the two interventions. (DOCX) [file pone.0198209.s002.docx]

**S2 Table A. Difference in mean population size between mixed age surgical sterilization and young age surgical sterilization interventions within the same level of surgical capacity. Level 1 represents a surgical capacity of 21 surgeries per month, level 2 represents 42 surgeries per month, and level 3 represents 84 surgeries per month. Percentages in brackets are the % reduction in mean population size between the two interventions.**

|  |  | **A. Mixed age surgical sterilization** | | |
| --- | --- | --- | --- | --- |
|  | Intervention number | A.1 | A.2 | A.3 |
| **B. Young age surgical sterilization** | B.1 | 1961  (-77.84%) | 1006  (-64.32%) | 66  (-10.58%) |
|  | B.2 | 2180  (-86.54%) | 1225  (-78.32%) | 285  (-45.67%) |
|  | B.3 | 2216  (-87.97%) | 1261  (-80.63%) | 321  (-51.44%) |

**S2 Table B. Difference in mean population size between mixed age surgical sterilization and female only mixed age surgical sterilization interventions within the same level of surgical capacity. Level 1 represents a surgical capacity of 21 surgeries per month, level 2 represents 42 surgeries per month, and level 3 represents 84 surgeries per month. Percentages in brackets are the % reduction in mean population size between the two interventions.**

|  |  | **A. Mixed age surgical sterilization** | | |
| --- | --- | --- | --- | --- |
|  | Intervention number | A.1 | A.2 | A.3 |
| **C. Female only mixed age surgical sterilization** | C.1 | 1987  (-78.88%) | 1032  (-65.98%) | 92  (-14.74%) |
|  | C.2 | 2174  (-86.38%) | 1219  (-77.94%) | 279  (-44.71%) |
|  | C.3 | 2284  (-90.67%) | 1329  (-84.97%) | 389  (-62.34%) |

**S2 Table C. Difference in mean population size between female only mixed age surgical sterilization and female only young age surgical sterilization interventions within the same level of surgical capacity. Level 1 represents a surgical capacity of 21 surgeries per month, level 2 represents 42 surgeries per month, and level 3 represents 84 surgeries per month. Percentages in brackets are the % reduction in mean population size between the two interventions.**

|  |  | **C. Female only mixed age surgical sterilization** | | |
| --- | --- | --- | --- | --- |
|  | Intervention number | C.1 | C.2 | C.3 |
| **D. Female only young age surgical sterilization** | D.1 | 225  (-42.29%) | 38  (-11.01%) | -72  (30.64%) |
|  | D.2 | 245  (-46.05%) | 58  (-16.81%) | -52  (22.13%) |
|  | D.3 | 256  (-48.12%) | 69  (-20.00%) | -41  (17.45%) |

**S2 Table D. Difference in mean population size between young age surgical sterilization and female only young age surgical sterilization interventions within the same level of surgical capacity. Level 1 represents a surgical capacity of 21 surgeries per month, level 2 represents 42 surgeries per month, and level 3 represents 84 surgeries per month. Percentages in brackets are the % reduction in mean population size between the two interventions.**

|  |  | **B. Young age surgical sterilization** | | |
| --- | --- | --- | --- | --- |
|  | Intervention number | B.1 | B.2 | B.3 |
| **D. Female only young age surgical sterilization** | D.1 | 251  (-44.98%) | 32  (-9.44%) | -4  (1.32%) |
|  | D.2 | 271  (-48.57%) | 52  (-15.34%) | 16  (-5.28%) |
|  | D.3 | 282  (-50.54%) | 63  (-18.58%) | 27  (-8.91%) |
