# Supplementary material for: Modeling the effect of surgical sterilization on owned dog population size in Villa de Tezontepec, Hidalgo, Mexico, using an individual-based computer simulation model
Source: PLoS One. 2018 Jun 1;13(6):e0198209. doi: 10.1371/journal.pone.0198209 (PMC5983437; doi:10.1371/journal.pone.0198209)
Supplement: S3 File — Figure A. Comparison of the impact of increasing the annual risk of non-age related mortality for adult confined dogs from 1.5% to 7.5% for the mixed age sterilization interventions at the lowest level of surgical capacity. Model outcomes demonstrated increased variability as the adult mortality rate increased. Figure B. Comparison of the impact of increasing the annual risk of non-age related mortality for puppies confined from 5% to 30% for the mixed age sterilization intervention at surgical capacity of 21 surgeries per month. Model outcomes demonstrated subtle changes in the relative impact of the interventions under a wide range (5% - 30%) of values for this parameter value; however, these changes introduced the greatest variability in outcome for the highest level of puppy mortality. Figure C. Projected impact of increasing the annual risk of non-age-related mortality for young confined dogs from 10% to 30% for the mixed age sterilization intervention (surgical capacity = 21 surgeries per month). Model outcomes appear highly sensitive to this parameter value with higher mortality rates associated with significant variability in model outcomes. Figure D. Comparison of the impact of increasing the annual risk of non-age-related mortality for young unconfined dogs from 10% to 60% on the outcomes for the mixed age sterilization intervention at the lowest surgical capacity. Increasing the mortality rate for this age group resulted in more variability in the model outcomes. Figure E. Comparison of the impact of increasing the annual risk of non-age related mortality for unconfined puppies from 5% to 50% for the mixed age sterilization at the lowest surgical capacity. Model outcomes appear relatively stable across a wide range of this parameter value. Figure F. Comparison of the impact of increasing the annual risk of non-age related mortality for adult unconfined dogs from 1.5% to 9% on the outcomes for the mixed age sterilization intervention at the lowest surgical [file pone.0198209.s003.docx]

## S3 Fig A. Comparison of the impact of increasing the annual risk of special cause mortality for adult confined dogs from 1.5% to 7.5% for the mixed age sterilization interventions at the lowest level of surgical capacity. Model outcomes demonstrated increased variability as the adult mortality rate increased.

## S3 Fig B. Comparison of the impact of increasing the annual risk of special cause mortality for puppies confined from 5% to 30% for the mixed age sterilization intervention at surgical capacity of 21 surgeries per month. Model outcomes demonstrated subtle changes in the relative impact of the interventions under a wide range (5% - 30%) of values for this parameter value, however these changes introduced the greatest variability in outcome for the highest level of puppy mortality.

## S3 Fig C. Projected impact of increasing the annual risk of special cause mortality for young confined dogs from 10% to 30% for the mixed age sterilization intervention (surgical capacity = 21 surgeries per month). Model outcomes appear highly sensitive to this parameter value with higher mortality rates associated with significant variability in model outcomes.

**S3 Fig D. Comparison of the impact of increasing the annual risk of special cause mortality for young unconfined dogs from 10% to 60% on the outcomes for the mixed age sterilization intervention at the lowest surgical capacity. Increasing the mortality rate for this age group resulted in more variability in the model outcomes.**

**S3 Fig E. Comparison of the impact of increasing the annual risk of special cause mortality for unconfined puppies from 5% to 50% for the mixed age sterilization at the lowest surgical capacity. Model outcomes appear relatively stable across a wide range of this parameter value.**

**S3 Fig F. Comparison of the impact of increasing the annual risk of special cause mortality for adult unconfined dogs from 1.5% to 9% on the outcomes for the mixed age sterilization intervention at the lowest surgical capacity. In general, the model outcome appears relatively robust to variability in this parameter value.**

**S3 Fig G. Comparison of the impact of increasing the annual risk of pregnancy of female unconfined dogs from 10% to 66% on the outcomes for the mixed age sterilization intervention at the lowest surgical capacity. Model outcomes were sensitive to this parameter value with increases in the pregnancy rate resulting in a less favourable intervention outcome.**

## S3 Fig H. Comparison of the impact of increasing the annual risk of pregnancy of female confined dogs from 10% to 40% for the mixed age sterilization interventions at a surgical capacity of 21 surgeries per month. Increasing the pregnancy rate to 0.4 resulted in far less change in the population size as a result of the intervention than at lower pregnancy rates.

**S3 Fig I. Comparison of increasing the community capacity community from 2924 dogs (Panel A – Baseline community capacity) to 4498 dogs (Panel B- Full Community Capacity), on the outcomes of mixed age and young female only surgical sterilization interventions at the lowest surgical capacity (21 surgeries per month). In general, model outcomes appear relatively robust to variability in this parameter value, especially when the intervention focuses on sexually immature female dogs exclusively.**

**S3 Fig J. Comparison of the impact of increasing the risk of special cause mortality for sterilized confined dogs from 1.5% to 3.6% on the outcomes for the mixed age sterilization intervention at the lowest surgical capacity. In general, model outcomes did not demonstrate considerable changes in the relative impact of the interventions across the range of values examined.**

**S3 Fig K. Comparison of the impact of increasing the risk of special cause mortality for sterilized unconfined dogs from 2.7% to 6.0% on the outcomes for the mixed age sterilization intervention at the lowest surgical capacity. In general, model outcomes did not demonstrate substantial changes in the relative impact of the interventions across the range of values examined.**

**S3 Fig L. Comparison of the percentage change in the risk of natural cause mortality for sterilized dogs from -20% to +20% on the outcomes for the mixed age sterilization intervention at the lowest surgical capacity. In general, model outcomes did not demonstrate drastic changes in the relative impact of the interventions across the range of values examined.**
